# The role of family and computer-mediated communication in adolescent loneliness

**DOI:** 10.1371/journal.pone.0214617

**Published:** 2019-06-05

**Authors:** Lindsay Favotto, Valerie Michaelson, William Pickett, Colleen Davison

**Affiliations:** 1 Department of Public Health Sciences, Queen’s University, Kingston, Ontario, Canada; 2 Kingston General Hospital Research Institute, Kingston, Ontario, Canada; 3 School of Religion, Queen’s University, Kingston, Ontario, Canada; Universita Cattolica del Sacro Cuore Sede di Roma, ITALY

## Abstract

Adolescence is a developmental phase in which feelings of loneliness often increase. It is also a time period during which computer-mediated communication (CMC) is frequently used by youth to communicate with their peers. Strong family relationships protect youth from experiencing a wide range of adversities and mental health problems, including loneliness, and yet use of CMC to contact peers may leave adolescents feeling disconnected and lonely while also limiting the amount of time they spend with their family. This study examines the association between CMC and feelings of loneliness among Canadian youth, with family communication explored as an effect modifier. The study base was the Canadian 2013–2014 Health Behaviour in School-aged Children study used in a cross-sectional analysis (N = 30117; grades 6–10). Random-effects multilevel Poisson regression methods were used to quantify risks for adolescent loneliness among daily vs. non-daily users of verbal CMC (e.g., Skype, phone calls), text/instant messaging and social media CMC with friends. Effect modification was tested via the inclusion of modelled interaction terms. Family communication quality moderated the relationship between daily CMC use and loneliness among Canadian youth. Among youth experiencing high relative quality of family communication, daily use of verbal and social media CMC to contact friends was positively associated with reports of loneliness, compared to non-daily users. Findings suggest that family communication must remain central in societal discussions of youth loneliness, mental health and use of CMC.

## Introduction

Feelings of loneliness are often heightened during adolescence; approximately 20% of Canadian adolescents self-identify as being lonely [1]. Seminal reviews have defined loneliness as feelings that result from the absence of a social life that one desires, including a perceived discrepancy between the social contacts one has in relation to what they crave, an increase in their need for social connection that is not met, or a subjective feeling of isolation regardless of surrounding social opportunities [2–6]. Loneliness is considered a significant risk factor for mental illness such as anxiety and depression [3,7–9], which is estimated to affect 13.4% of youth worldwide [10]. Thus, evidence regarding contemporary factors, such as social media use, that contribute to the development of loneliness is of great importance to the prevention of mental illness among youth.

The ways in which youth socialize and build relationships is now strongly driven by computer-mediated communication (CMC), which involves communication with others through or by use of computers or the Internet [11,12], as many of their interactions with their peers occur through digital means [13–16]. In the United States, 92% of youth report being online daily [13]. Canadian youth send an average of 67 text messages per day [13], and 89% indicate that they use at least one social media platform [17]. Even though use of CMC can help to maintain communication during breaks in physical presence [18] and expand social circles [19], there is evidence to suggest that use of CMC reduces social support [20], quality of relationships and empathy for others [21], and time spent face-to-face with friends [22]. Given that youth are at high risk for feelings of loneliness, their use of different forms of CMC to communicate with their peers has the potential to modify these feelings.

Strong family relationships protect youth from experiencing a wide range of adversities and mental health problems [23]. Canadian data show that 71–80% of Canadian youth report feeling trusted by their parents and 67–84% report having a happy home life [24], which suggests that most Canadian youth experience strong relationships with their family. The link between family support and adolescent mental distress has been established; however, little evidence exists regarding the role of the family in the relationship between CMC and loneliness. Previous research has identified a negative association between CMC and time spent face-to-face with family [20,25,26]. Thus, the use of CMC and reduced family interaction may negatively impact social connectedness and increase risks of loneliness among youth. Interactions between CMC with peers, family communication and precursors to the development of mental health problems, such as loneliness, have not been well studied in adolescent populations. Given the reality of our technology-rich world, and since youth continue to adopt increasingly diverse CMC methods [17], it is imperative that we understand such questions.

We had the opportunity to employ data from a national and contemporary study to examine associations between CMC and feelings of loneliness among Canadian youth. Quality of family communication was considered as a potential effect modifier in our models. Based on the established association between strong family relationships and positive health outcomes [23,27,28], we speculated that engagement in various methods of CMC (e.g., internet, messaging) for daily contact with friends, as well as low quality of family communication, would each increase feelings of loneliness. Additionally, given the link between CMC use and reduced family communication [20,25,26], we hypothesized that family communication would modify the relationship such that low family communication would increase the risk for loneliness more strongly among daily users of CMC compared with non-daily users.

## Methods

### Participants

This study was based on the 2013–2014 (cycle 7) of the Canadian Health Behaviour in School-aged Children (HBSC) study [24]. The HBSC study involves 44 countries and regions, including Canada, and is conducted every four years in collaboration with the World Health Organization [24]. The focus population is young people aged 11 to 15 years. In Canada, the target population is students in grades 6 to 10 [24]. Canadian samples are obtained using a multi-stage cluster sampling method. The primary sampling units are school classrooms nested within schools and associated school boards [24,29]. For the sample to represent the Canadian population of young people, these data are weighted to adjust for over and under sampling at the provincial and territorial level [24]. The 2013–2014 cycle included 30,117 students, before the application of survey weights, from 377 schools across Canada [24]. Overall, this cycle attained a 100% response rate at the provincial/territorial level, 50.3% response rate at the school level and a 77% participation rate at the level of individual students [24]. After removal of missing data, the final sample included 23,218 Canadian youth before application of survey weights.

### Measures

#### Outcome: Self-perceived loneliness

Youth completed the HBSC survey item: "I often feel lonely?" (5-point Likert scale from “strongly disagrees” to “strongly agree”). This individual item is part of the Multidimensional Scale of Perceived Social Support [30], which has been used extensively in past studies [24,31] and has undergone tests for validity to subjectively assess feelings of loneliness in Canada and elsewhere [31]. For this study, responses were dichotomized, and often feeling lonely was defined as those reporting “strongly agree” or “agree” to the above item.

#### Exposure: Computer-mediated communication use

CMC variables were quantified as: (a) phone calls, and use of Skype or FaceTime, (b) instant messaging (BBM, Facebook chat), (c) text messaging, (d) email and, (e) social media (Facebook [not including chat], My Space, Twitter, Apps [Instagram], games [Xbox], YouTube, etc.). For this study, CMC was defined as four separate exposures including: (1) verbal CMC (phone calls, Skype and FaceTime), (2) messaging CMC (instant messaging and text messaging, Cronbach’s α = 0.72), (3) social media CMC and (4) email use. These items have established reliability: verbal CMC r = 0.52 (p < .001), messaging and SMS CMC r = 0.76 (p < .001), instant messaging CMC r = 0.75 (p < .001), and social media CMC r = 0.64 (p < .001) along with correlation to the previous HBSC CMC item: verbal CMC r = 0.48 (p < .001), messaging and SMS r = 0.71 (p < .001), instant messaging r = 0.63 (p < .001), and social media r = 0.52 (p < .001) [32]. For all CMC measures, “daily” use was separated from “non-daily” use to create a dichotomous variable, consistent with past precedents [24].

#### Effect modifier: Family communication quality

The four-item communication scale (Cronbach’s α = 0.88) of the Family Dynamics Measure II scale [33] was used. Sample items include, “when I speak someone listens to what I say” and "when there is a misunderstanding we talk it over until it's clear." Item responses range in five categories from “strongly agree” to “strongly disagree.” Based on the distribution of the data, the family communication scale was divided into quartiles for the analysis. The scale has shown strong internal consistency among students aged 11–17 (Cronbach’s α = 0.82), and their parents (Cronbach’s α = 0.85) [34].

#### Potential confounders

Age [35,36], sex [35,36], family characteristics such as family structure [27] and family income [7,37], presence of mental health concerns [38], and peer characteristics such as perception of peer support, frequency of face-to-face interactions and involvement in group activities were identified as potential confounders due to there identified link to both loneliness and CMC use [35,37].

Family structure was identified by asking with whom the participant lives with most of the time [32]. A dichotomous variable was created that identified nuclear (e.g., living with both mother and father) or non-nuclear family structures, consistent with precedent [39].

To assess family wealth, the HBSC includes a proxy item that asks students to indicate how well off they perceive their family to be ("very well off" through "not well off at all"). This item generates a measure of perceived relative socioeconomic position that has strong test-retest reliability and correlates to household income and parental education [40].

Quality of relationships with peers was measured using the four-item peer support sub-scale (Cronbach’s α = 0.92) of the Multidimensional Scale of Perceived Social Support (MSPP), which was developed to quantify the subjective experience of social support [41]. Youth reported on their perceived social support using a 5-point Likert scale. This measure has been psychometrically tested among adolescents and university students with good inter-rater reliability (0.75) [41] and internal consistency (Cronbach’s α = 0.90) [30,32].

To assess peer contact, participants were asked how often they meet their friends outside of school time during two-time points in the evening (before and after 8:00 p.m.) which has been used in past studies [35] and has high reliability (Cronbach’s α = 0.73).

Involvement in group activities was defined by an indication of participation in any of six different group activities (i.e. team sport, volunteer work, arts or community group along with, “other” activities or group involvement). Following precedent [42], the overall level of involvement was divided into categories: (a) no involvement, (b) one group activity, (c) two group activities, (d) three or more group activities.

The presence of mental health issues was assessed using one validated item taken from the Diagnostic Interview for Children Version IV (DISC-IV) [43]. Youth were asked, “during the past 12 months, did you ever feel so sad or hopeless almost every day for two weeks or more in a row that you stopped doing some usual activity.” This item has strong test-retest reliability amongst youth with diagnosed depression for at least one year (k = 0.92) [43] and is commonly used as an initial screen for suicidal ideation [43].

### Data analyses

Frequency distributions of key exposures, the study outcome and potential confounders were examined. Independent associations between the outcome of loneliness and all exposure variables of interest were tested via the Rao-Scott Chi-square test for complex survey data after transforming scales into categorical variables [44]. To identify the level of variance in loneliness that occurred at the group level of each school, the intraclass correlation coefficient (ICC) was estimated using the null (empty) model [45]. The ICC indicated that 1.6% of the variance of loneliness was attributed to between school differences [45]. Even with such limited levels of clustering, we adhered to random-effects multilevel modelling to adjust for the nested nature of data collection. A Multilevel Poisson Regression [46] was applied with a log link function and application of sample weights. Each model quantified the relative risk (RR) and 95% confidence interval (CI) for each association. Modelling was initiated with unadjusted models of each exposure to the outcome of loneliness, followed by models that considered the addition of potential covariates.

Effect modification by family communication quality was assessed through the insertion of a two-way multiplicative interaction term in each model. The most parsimonious, conservative models were identified through stepwise selection procedures with the selection criteria of p < .20 for potential confounders [47]. Assessment of confounding was also considered through the change in estimate approach [47]. Power calculations [48] were conducted for all three CMC exposures, sub-group absolute differences of 2% to 4% were detectable with 80% power, with consideration of effect modification.

With limited missing data (~10% across all variables), and the frequency of loneliness and daily use of CMC remaining constant after removal of respondents with any missing data for exposure, outcome or covariate items, a complete case analysis was conducted [49]. Removal of individuals with missing variables did result, however, in a non-significant increase in proportion of females (52.6% vs. 50.7%, p = .32) and grade 9 to 10 students (48.1% vs. 44.4%, p = .26) in the final sample.

All statistical procedures were conducted using SAS software version 9.4 (Cary, NC: SAS Institute Inc.). The Queen’s University Research Ethics Board, the Public Health Agency of Canada and Health Canada’s Research Ethics Board approved the 2014 Canadian HBSC study. Further, the Queen’s University Health Sciences Research Ethics Board approved this specific study (EPID-520-15; Romeo approval number #6016097).

## Results

Characteristics of the sample are described in Table 1. The prevalence of self perceived loneliness was 24.6% (95% CI = 23.6%-25.7%) and was reported more often by female (29.9%, 95% CI = 28.4%-31.3%) compared to male (19.0%, 95% CI = 17.8%-20.2%) respondents (Table 2). Daily use of all methods of CMC, except email, was associated with greater reports of loneliness (p < .05). Lower relative levels of family communication quality were also associated with a greater proportion of reported loneliness (p < .0001).

**Table 1:** Description of the Canadian sample population in the 2013–2014 HBSC study^a^ (N = 30117).

Characteristic	n	%	Characteristic	n	%
Sex			Perceived Family Wealth		
Male	14784	49.3	High	15120	53.5
Female	15178	50.7	Average	10456	37.0
			Low	2703	9.6
Family Communication Quality			Involvement in Group Activities		
Q1 (highest)	6303	22.3	3+	8313	29.7
Q2	9554	33.9	2	7397	26.4
Q3	5333	18.9	1	8232	29.4
Q4 (lowest)	7012	24.9	None	4071	14.5
School Grade			Primary Outcome: Loneliness		
6–8	16273	54.6	No	20735	73.8
9–10	13520	44.4	Yes	7376	26.2
Feeling Sad or Hopeless			Exposure 1: Email Use		
No	20489	71.6	Non-daily User	26549	97.5
Yes	8118	28.4	Daily User	681	2.5
Family Structure			Exposure 2: Verbal CMC		
Nuclear Family	19581	69.0	Non-daily User	20984	74.1
Non-Nuclear Family	8796	31.0	Daily User	7338	25.9
Frequency of Contacting Friends			Exposure 3: Messaging CMC		
Weekly or more	13688	50.9	Non-daily User	14897	53.7
Less than weekly	13204	49.1	Daily User	12856	46.3
Peer Support			Exposure 4: Social Media CMC		
High	22896	83.6	Non-daily User	19243	68.7
Low	4496	16.4	Daily User	8784	31.3

^a^HBSC sample before application of survey weights

**Table 2:** Prevalence of loneliness by socio-demographic, family and peer characteristics (row%)^a^ (N = 30117).

Characteristic		n	Lonely (n = 6897)		p-value^b^
			n	row% (95% CI)	
Sex					
	Male	13466	2562	19.0 (17.8–20.2)	< .0001
	Female	14438	4313	29.9 (28.4–31.3)	
Family Communication Quality					
	Q1 (highest)	6,499	855	13.1 (11.6–14.7)	< .0001
	Q2	9,380	1641	17.5 (16.2–18.8)	
	Q3	4,960	1305	26.3 (24.2–28.4)	
	Q4 (lowest)	6,459	2914	45.1 (43.1–47.1)	
School Grade					
	6–8	15153	3263	21.5 (29.3–22.8)	< .0001
	9–10	12555	3524	28.1 (26.7–29.4)	
Feeling Sad or Hopeless					
	Yes	7404	3964	53.6 (51.3–55.7)	< .0001
	No	19568	2651	13.5 (12.7–14.4)	
Family Structure					
	Nuclear Family	19330	4174	21.6 (20.5–22.7)	< .0001
	Non-Nuclear Family	7984	2545	31.9 (30.1–33.6)	
The frequency of Contacting Friends					
	Weekly or more	12303	2809	22.8 (21.5–24.1)	< .0001
	Less than once per week	13781	3627	26.3 (24.9–27.7)	
Peer Support					
	High	22900	5108	22.3 (21.1–23.5)	< .0001
	Low	4381	1560	35.6 (33.0–38.2)	
Perceived Family Wealth					
	High	15138	2870	19.0 (17.9–20.1)	< .0001
	Average	9337	2702	28.9 (27.3–30.5)	
	Low	2517	1086	43.1 (39.5–46.7)	
Involvement in Group Activities					
	3+	7126	1810	25.4 (24.0–26.8)	< .0001
	2	7503	1620	21.6 (19.9–23.2)	
	1	8403	1893	22.5 (21.0–24.0)	
	None	4317	1417	32.8 (30.2–35.5)	
Email Use					
	Non-daily User	25715	6293	24.5 (23.4–25.6)	.4594
	Daily User	778	206	26.5 (21.1–31.8)	
Verbal CMC					
	Non-daily User	20338	4859	23.9 (22.7–25.0)	.0017
	Daily User	6914	1832	26.5 (24.9–28.1)	
Messaging CMC					
	Non-daily User	14188	3308	23.3 (22.0–24.7)	.0023
	Daily User	12655	3302	26.1 (24.7–27.5)	
Social Media CMC					
	Non-daily User	18276	4267	23.3 (22.2–24.5)	< .0001
	Daily User	8871	2415	27.2 (25.5–29.0)	

Notes: CMC, computer-mediated communication

^a^ Adjusted for school-level clustering and weighted for population representativeness

^b^ p-value for the Rao Scott chi-square test between each characteristic and loneliness

Family communication quality was found to modify the association between loneliness and daily use of verbal and social media CMC (Tables 3 and 4). The interaction was explored in two ways: (1) identifying the risk of loneliness between levels of family communication quality, within daily and non-daily users of CMC, independently (Table 3), and (2) quantifying the risk of loneliness between daily and non-daily users within each quartile of family communication (Table 4).

**Table 3:** The association between family communication quality and loneliness by non-daily and daily CMC use as reported by Canadian adolescents (n = 23,218).

	RR_adj_ (95% CI) of Loneliness by Quartile of Family Communication Quality^a^^,^^b^				
Frequency of CMC Use	Q1 (highest)	Q2	Q3	Q4 (lowest)	P-value for interaction^c^
Verbal CMC					
Non-daily User	1.00	1.39 (1.24–1.55)*	1.77 (1.57–1.98)*	2.20 (1.98–2.45)*	.011
Daily User	1.00	0.98 (0.82–1.17)	1.37 (1.15–1.65)*	1.75 (1.49–2.05)*	
Messaging CMC					
Non-daily User	1.00	1.35 (1.19–1.54)*	1.79 (1.56–2.05)*	2.19 (1.93–2.48)*	.201
Daily User	1.00	1.13 (0.99–1.30)	1.47 (1.28–1.69)*	1.92 (1.69–2.17)*	
Social Media CMC					
Non-daily User	1.00	1.36 (1.21–1.53) *	1.82 (1.61–2.05)*	2.23 (1.99–2.50)*	.008
Daily User	1.00	1.05 (0.91–1.22)	1.31 (1.12–1.54)*	1.71 (1.49–1.96)*	

Note: CMC, computer-mediated communication

^a^ Adjusted for school-level clustering and weighted for population representativeness.

^b^ Multilevel Poisson regression controlling for sex, school grade, feeling low or depressed, family structure, the frequency of face-to-face contact with peers, involvement in group activities, perceived family wealth, and peer support.

^c^ Interaction of CMC*family communication quality.

* p < .05

**Table 4:** The association between non-daily and daily CMC use and loneliness by each quartile of self-perceived family communication quality as reported by Canadian adolescents (n = 23,218).

	RR_adj_ (95% CI) of Loneliness by Frequency of CMC User^a^^,^^b^			
Quartile of Family Communication Quality	Non-daily User	Daily Verbal CMC User	Daily Messaging CMC User	Daily Social Media CMC User
Q1 (highest)	1.00	1.29 (1.09–1.52)*	1.11 (0.96–1.30)	1.34 (1.15–1.56)*
Q2	1.00	0.91 (0.80–1.03)	0.93 (0.84–1.03)	1.05 (0.94–1.17)
Q3	1.00	1.00 (0.87–1.15)	0.92 (0.81–1.03)	0.98 (0.86–1.11)
Q4 (lowest)	1.00	1.02 (0.93–1.11)	0.98 (0.90–1.06)	1.03 (0.95–1.12)
Interaction p-value^c^		**.009**	.201	**.012**

Note:

^a^ Adjusted for school-level clustering and weighted for population representativeness.

^b^ Multilevel Poisson regression controlling for sex, school grade, feeling low or depressed, family structure, the frequency of face-to-face contact with peers, involvement in group activities, perceived family wealth, and peer support.

^c^ Interaction of CMC.

* p < .05

Table 3 describes the relationship between loneliness and family communication quality stratified by non-daily and daily CMC users. Among both non-daily and daily users of verbal CMC (p = .01) and social media (p = .008) CMC, lower relative levels of family communication (Q2-Q4) were positively associated with loneliness compared to high levels of family communication. Therefore, the association between family communication and loneliness increases as family communication levels reduce, regardless of CMC use.

Table 4 further probes the interaction between CMC use and family communication quality by identifying the risk of loneliness within each quartile of family communication, comparing youth who use CMC daily to non-daily users. In environments of poor quality of family communication (Q2-Q4), daily use of all types of CMC showed little to no association with loneliness (p >.05). Conversely, among youth experiencing high relative quality of family communication (Q1), daily use of verbal and social media CMC to contact friends was positively associated with reports of loneliness, compared to non-daily users (RR_verbal,Q1_ = 1.29, 95% CI = 1.09–1.52). Overall, there was little difference in the magnitude of the association between all types of CMC.

## Discussion

This study explored associations between the use of verbal, text messaging and social media CMC to contact friends and self-perceived loneliness in Canadian young people along with effect modification by the quality of family communication. We found that the prevalence of loneliness was higher for daily CMC users compared with non-daily users of CMC, irrespective of the type of CMC used. Further, family communication quality modified the relationship between verbal and social media CMC use and loneliness such that, among families of high-quality communication, youth report greater loneliness with daily use of CMC.

Overall, the relationship between CMC use and loneliness did not strongly differ by the method of CMC used. Therefore, even though there are distinct differences between the methods of communication among all types of CMC investigated in this study, all types of CMC have a positive relationship with loneliness. We did observe differences in modification of family communication on the association between loneliness and verbal and social media CMC. These differences could be explained by lack of power to detect effect modification or the higher value that youth place on their relationships with individuals that they communicate with via social media CMC [50]. Thus, daily use of social media CMC may further take youth away from engaging with their family. Furthermore, the visual and interactive nature of verbal and social media CMC may facilitation a greater perceived discrepancy between the social interactions that youth have and that which they hope to have.

Findings of this study are consistent with recent systematic reviews [51,52] that highlight the association between CMC and loneliness, along with anxiety and depression. This observed association could be explained through the features of CMC that are inherently different from face-to-face contact [11,53]. The impersonal features of CMC, such as little to no eye contact and a lag time in message response [11,13], may prevent meaningful and rich conversations [54], contributing to feelings of loneliness. Additionally, a bidirectional relationship may also explain these findings as it is suggested that socially isolated youth may utilize CMC methods to compensate for offline social relationships [55]. One important insight that this current study contributes to this discourse is the nuanced consideration of the role of family communication in relation to CMC and loneliness.

Not surprisingly, poor quality of family communication remains a strong predictor of youth loneliness. Consistent with previous findings [27], youth gain significant social support from their family during early adolescence. Without high quality of family communication, youth may perceive a discrepancy in the quality of interactions that they hope to have, or which they do have with their family members, which in turn may lead youth to self-identify as being lonely [56]. Given the salient influence of family during this period of youth development and how family relationships predict loneliness among this age group, family-centered interventions may be an essential avenue to support the psychological well-being of youth to ameliorate feelings of loneliness.

Among youth experiencing high relative quality of family communication (Q1), daily use of CMC to connect with friends was associated with greater reports of self-perceived loneliness after considering other factors such as age and sex. In other words, high quality of family communication protects youth from loneliness, but if they use CMC devices daily, they report greater feelings of loneliness. This finding is unexpected as a strong parent-child relationship is known to reduce psychiatric risk [57,58]. Further, this finding directly contradicts the conclusions made in previous literature [27] but can be explained through the use of the displacement theory. This theory proposes that use of CMC displaces time spent otherwise engaged in other activities such as face-to-face communication [20,59]. Therefore, the use of CMC may take away from time spent with their family as youth choose to utilize their time to engage in CMC with their peers. Findings suggest that youth report high quality of family communication but still experience loneliness from reduced quantity of family interactions due to their CMC use with friends. This displacement may only negatively influence the youth of high relative family communication because they would otherwise gain support and protection from loneliness by interacting with their supportive family. Others with lower levels of family communication do not have this supportive environment available to them; therefore the use of CMC may not negatively impact their reports of loneliness.

Considering the limitations of this study is important. First, we were unable to create a strong CMC exposure gradient, as the extent of daily use was not available for analysis. Second, potential confounders, such as personality traits, could have been missed resulting in uncontrolled confounding in the analysis. Third, the HBSC is a cross-sectional survey; therefore causation or directionality cannot be inferred. Last, our large sample size allows for the detection of even small effects, but these results are also practically important as they are consistent with previous literature [20–22]. Strengths of this study include the depth and timely nature of the analysis. Further, the weighted HBSC data allows for findings to be based on a national representation of Canadian youth. Last, the HBSC survey assesses the use of different CMC methods, beyond overall internet use, allowing for insight into the different CMC methods utilized by Canadian youth.

Future research should look at these associations using the frequency of CMC within a day and how youth are using CMC within older populations of youth, which may utilize CMC to communicate with peers at a higher frequency. This would aid in further understanding the social implications of CMC use among different aged youth, and among young people who use different types of CMC and at different frequencies.

## Conclusions

In a highly-digitalized world in which many young people learn to socialize using CMC, identifying and understanding the implications of using these devices is essential. The findings of this study indicate that the use of CMC is associated with greater reports of loneliness among youth, even within families with strong communication. Thus, communication through computed-mediated means may not facilitate the same social support and connection as face-to-face communication.
